# Development of a comprehensive assessment model for coral reef island carrying capacity(CORE-CC)

**DOI:** 10.1038/s41598-021-83481-w

**Published:** 2021-02-16

**Authors:** Ming Chen, Fenzhen Su, Fei Cheng, Yu Zhang, Xuege Wang

**Affiliations:** 1 State Key Laboratory of Resources and Environmental Information System, Institute of Geographic Sciences and Natural Resources Research, Chinese Academy of Sciences, Beijing, 100101 China; 2 College of Resources and Environment, University of Chinese Academy of Sciences, Beijing, 100049 China; 3 School of Geography and Ocean Sciences, Nanjing University, Nanjing, 210023 China

**Keywords:** Environmental social sciences, Sustainability

## Abstract

Coral reef islands provide precious living space and valuable ecological services for human beings, and its sustainability cannot be ignored under the pressure of human activities. Carrying capacity (CC) assessment has gradually become an important means to measure sustainability of islands. However, there is little comprehensive evaluation of the carrying capacity of coral reef islands, and traditional evaluation methods are difficult to express the social-ecological characteristics of coral reef islands. The present paper proposes a comprehensive assessment model for coral reef island carrying capacity (CORE-CC) which comprises dimensions of resources supply, environmental assimilative, ecosystem services, and socio-economic supporting. According to the characteristics of the coral reef islands, the core factors and indicators of each dimension are selected and the corresponding assessment index system of "pressure-support" is constructed. The assessment involves (1) identification of carrying dimensions and core factors, (2) pressure/support measurement and (3) assessment of carrying state. A case study is conducted in Zhaoshu Island of China, demonstrating the applicability of CORE-CC model and serving as a reference for adaptive management.

## Introduction

Coral reefs are among the richest and most diverse ecosystem types found in the global ocean^1,2^. Coral reef islands are formed by biological debris produced by the erosion, deposition and cementation of coral sediments. As a special type of islands, coral reef islands not only provides the carrier for human habitation and the fulcrum for protecting and utilizing the ocean, but also provides valuable ecosystem services, such as food provision, coastal protection, recreational activities and numerous cultural services^3,4^. However, due to the effects of local human activities, such as overfishing, land runoff, and pollution discharge, coral reefs’ sustainability is questioned around the world^5^. Coral reef systems are social-ecological systems where the interaction between humans and nature is particularly complex^4^. Human beings are the main body in the development and utilization of coral reef resources, and at the same time they bring threats and damage to coral reef ecosystems, but they are also the promoters of the protection and management of coral reef environment^6^. However, the management of the ecological environment should not only be passive restoration, active adaptive management to maintain the health of the ecosystem is more important^7^. The core task of adaptive management is to identify and analyze the basic resource advantages and stress factors of the regional ecosystem, and propose management models and countermeasures on this basis^8,9^. Currently, some scholars tried to achieve sustainable development of coral reef social-ecological system by revealing the interactions among socio-economic and ecological environment factors or addressing the stressors and the impact pathways, few of which have applied the carrying capacity theory in coral reef islands^10–13^. Carrying capacity is a scientific concept for measuring the relationship between human socio-economic activities and the natural environment, and an important tool for measuring and managing human sustainable development^14^. Meanwhile, the carrying capacity analysis method combines qualitative comparison and quantitative evaluation, which can reveal the stress factors and characteristics of the regional ecosystem to a certain extent, and can provide a basis for adaptive management^15^. Carrying capacity can be an effective perspective to measure the sustainability of coral reef islands^16^.

The concept of carrying capacity (CC) derives from a demographic focus on the sustainable growth of population or livestock farming, this concept demonstrates the relationship between a population (carrying object) and the environment (carrier)^17^. Accompanied with the development and enlarging range of human activities, the research on carrying capacity has changed from researches of biotic population growth law to the comprehensive carrying capacity covering natural resource endowments and human development demands^18^^.^ The scopes of carrying capacity applications have been expanded, such as land resource carrying capacity^19,20^, water resource carrying capacity^21,22^, environmental carrying capacity^23,24^, ecological carrying capacity^25,26^, social carrying capacity^27,28^ and comprehensive carrying capacity^29–31^. The progression of carrying capacity has gradually attached importance to the influence of human activity factors on the carrying capacity^19^. The evaluation object has gradually shifted from a single resource and environmental element to the carrying capacity for multiple or comprehensive elements^32^.

With the development of the marine economy, human activities, coastal development and pollution have gradually changed and destroyed the capacity of marine ecosystems^2,33^, the researches on comprehensive assessment of CC are also applied to various fields of the ocean, such as Marine ecosystem, coastal zone and islands, etc^34–37^. However, as one of the most important marine ecosystems and type of islands, case studies on coral reef island carrying capacity are still limited to a single capacity, for instance, diving tourism carrying capacity or aquaculture carrying capacity^38–40^. The social-ecological system of coral reef islands is complex, we need to comprehensively assessment its carrying capacity to better reflect the entire system’s capacity.

Fortunately, the assessment methods of ordinary islands provide a reference for the comprehensive assessment of coral reef islands CC. These methods mainly include the system dynamics model^41^, ecological footprint method^42^, energy analysis^43,44^, and the comprehensive indicator system^45,46^. The system dynamics model can quantitatively analyse the intrinsic relationship between the structure and function of various complex systems; however, it is difficult to select the parameter indicator, and the large quantity of data limits the application of the model and the similitude to reality^47^. Both the ecological footprint method and the emergy analysis method simplify the complicated processes by an equivalent term which can neither tell considerable information relevant to the complexity of coupled social-ecological systems nor sufficiently feature a specific area^48^. Two common comprehensive indicator systems are as follows: one is to use a multi-dimensional indicator system, select indicators from the dimensions of resources, environment, ecology, socio-economic and assume ideal values for comprehensive evaluation; the other aims to build a PSR (Pressure-State-Response) or DPSIR (Driving-Force-Pressure-State-Impact-Response) model as a system layer or distinguish between pressure indicators and support indicators^49,50^.

Although the comprehensive indicator system method is more flexible and more in line with the actual situation^51^, some limitations exist. Logic feedback frameworks based on PSR or DPSIR are usually applied in qualitative evaluation due to the difficulties in data collection and weight determination^52^. For the multi-dimensional indicator system method, most studies divide the carrying capacity system into a resource standard, environmental standard and human society standard, and calculate the indexes of each standard layer as a whole. However, the ultimate comprehensive research results are difficult to judge whether the examined carrying capacity is overloaded^50^. Recently, many scholars believe that it is necessary to analyze the relationship between the support of the carrier and the pressure carried by the carrier to reflect the carrying capacity, so that the state of the carrying capacity can be more accurately judged^53–55^. But the evaluation objects are mostly single elements, such as water resources^53^, ecological environment^54^, etc., which can only provide partial understanding of sustainability^55^. Therefore, we can combine the multi-dimensional indicator system method and pressure-support analysis method to evaluate the carrying capacity more comprehensively and accurately. Meanwhile, in view of the commonness of the islands and the uniqueness of the coral islands in the ecological dimension, the evaluation index system and methods for the coral reef island CC should be improved accordingly.

To address these shortcomings, this paper presents a comprehensive assessment model of coral reef island carrying capacity (CORE-CC), which is composed of four dimensions: resources supply, environmental assimilative, ecosystem services and socio-economic supporting. Comprehensively evaluates the carrying state according to the corresponding relationship between the pressure and support capacity of the same core indicators. Finally, a case study is conducted in a typical coral reef island of Zhaoshu, and serves as a reference for local adaptive management.

## Methods

### CORE-CC conceptual model

**Figure 1:**
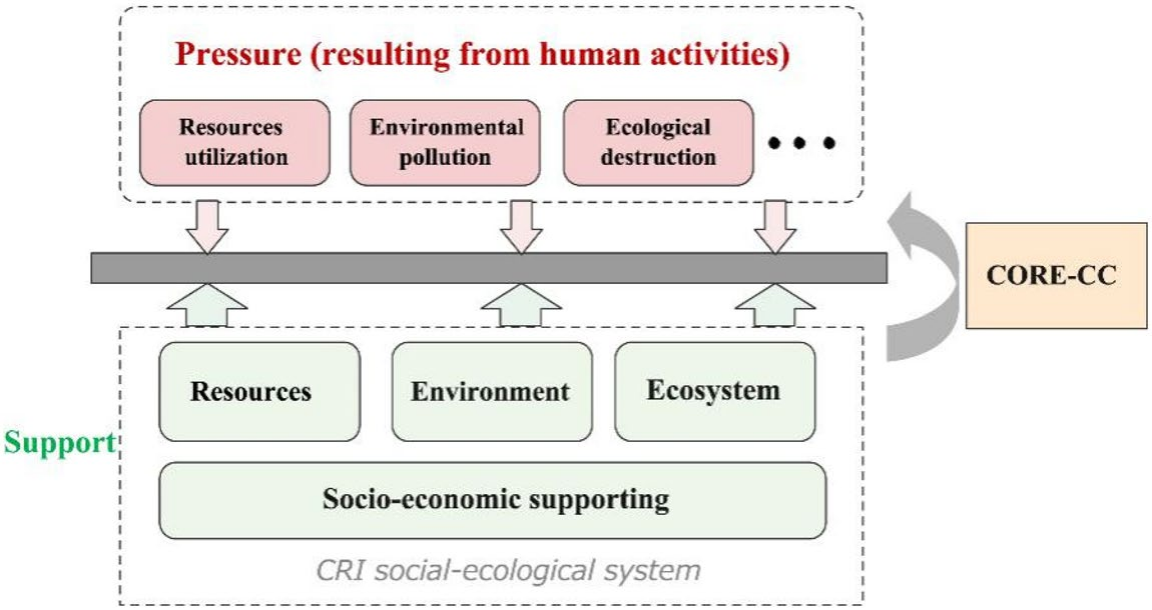
CORE-CC conceptual model. The ellipsis in the pressure box represents other human activities that interact with nature and exert pressure on the social-ecological system.

As a primary depict, Carrying capacity can be regarded as the limit of human activities that will cause adverse changes in the environment, provided that the environment itself imposes certain restrictions on development^56^. Therefore, it is necessary to understand the conflict between the limited support capacity of the coral reef island system and the pressure caused by the increase in population and human activities^57,58^. In other words, it is essential to check whether population and human activities exceed the capacity provided by coral reef Island to achieve the mission of sustainable development. However, the pressure brought by human activities on coral reef islands is reflected in many aspects, such as resources extraction and utilization, pollutant discharge, ecological damage caused by excessive access to ecosystem services, etc. Correspondingly, the coral reef islands system provides supports of resource supply, environmental assimilation and ecosystem service to cope with pressures. Although human socio-economic development is the source of pressures, it can improve the structure and function of the regional ecological environment through economic investment and technological innovation to increase the carrying capacity. Thus the essence of coral reef islands carrying capacity (CORE-CC) is kind of comparison between the pressure caused by human activities and the support capacity of the coral reef islands system, covering the four dimensions of resource, environmental, ecological, and socio-economic (Fig. 1). In this perspective, as long as the pressure is within the support capacity threshold, the coral island system can remain healthy and stable^35,53,54^. Herein, CORE-CC is defined as the combined threshold of resources supply, environmental capacity, ecosystem services, and socio-economic supporting capacity of the coral reef islands that could carry socio-economic activities without causing degradation of the structure and function of the social-ecological system.

### CORE-CC dimensions and their core factors

The above definition implies that CORE-CC has four dimensions: resources supply, environmental capacity, ecosystem services, and socio-economic supporting. Basically comprehensively expressed the scope of interaction between human activities and the ecological environment of coral reef islands. However, due to the differences in geographical environment, coral reef islands are considered to have different natural capital stock changes and utilization patterns, resulting in serious vulnerability, monomer structure, development differences and coral dependence and other unique economic characteristics^59^.

As a result, it leads to differences in the economic structure and functional orientation of different coral reef islands, the types and quality of factors that CORE-CC focuses on in each dimension are also different. Therefore, it is more meaningful to select the core factors of each dimension of CORE-CC according to the common characteristics of coral reef islands. The coral reef islands are common characterized by: (a) insularity, i.e. small size, limited water resources and heavily dependent on imports problems^60^; (b) human presence and shaping of landscapes for long time periods, leading to the development of semi natural environment to urbanization with the changes of economy and society^61,62^; (c) economy dominated by tourism, services and fisheries^62,63^; (d) it has both terrestrial and marine ecosystems, as well as a unique coral reef ecosystem^16^. These characteristics lead to the identification of the core factors for each dimension of CORE-CC. In addition, the word "CORE" means that the model considers the core factors of each dimension.

1. The resources supply dimension refers to the ability of the reef islands ecological environment to provide natural resources for human activities. Coral reef islands can provide humans with resources such as land, water, food, and a small amount of fuel^64^. Among them, water is a precious source of life^65^, land resources provide a foothold for human survival^66^, and most coral reef islands rely on imports for food and fuel^64^. Considering the scarcity and importance of resources, ensuring the sustainability of water and land resources is the focus of this research^67^. Therefore, Land resources and water resources are used herein as the core factors to measure the resources supply capacity in CORE-CC.
2. The environmental assimilation dimension refers to the migration of the reef islands environment and the self-purification function of transforming pollutants. The environmental assimilation of the coral reef islands should theoretically include the water assimilation, atmospheric assimilation and soil assimilation^56^. However, coral reef islands have few large industries, and at the same time, due to the fast air circulation of the coral reef islands, makes air pollution is easy to reduce. Furthermore, scarce land resources are usually not used as pollutant discharge carriers, and the seawater around the island is the main receptor for pollution discharge^68^. Therefore, the water assimilation is used herein as the core factor to measure the environmental assimilative capacity.
3. The ecosystem services dimension refers to the ability of ecosystems within a certain time and space to provide services such as ecological regulation, ecological support, and ecological culture^69^. Coral reef islands have the characteristics of land-sea transition patches, and their ecosystem service capacity should be considered from both terrestrial ecosystem and marine ecosystem^70,71^. Among them, the marine is dominated by coral reef ecosystem services, which is also a unique feature of the CORE-CC.
4. The socio-economic supporting dimension refers to the ability of human socio-economic to improve the resources supply capacity, environmental assimilative capacity and ecosystem services capacity of the coral reef islands. Human socio-economic's support behavior includes research and development of more resources, pollution control, environmental construction, ecological protection and restoration construction, etc^35,54^. Its capacity depends on the level of socio-economic development and the degree of support for the sustainable development of coral reef islands^51,72^. Therefore, the socio-economic supporting capacity is measured using socio-economic development and protective initiatives as the core factors.

According to the analysis conducted above, the core factors of each dimension of CORE-CC are determined (Table 1). In the next step, according to the characteristics of each factor, the quantitative indicators are selected in accordance with the basic principles of regionality, representativeness, ease of operation, and symmetry of "pressure-support".

**Table 1:** Core factors of each dimension of CORE-CC.

Objective	Dimensions	Core factors
CORE-CC	Resource supply	Land resources
		Water resources
	Environmental assimilation	Water assimilation
	Ecosystem services	Terrestrial ecosystem services
		Coral reef ecosystem services
	Socio-economic supporting	Socio-economic development
		Protective initiatives

### Measuring indicators of each core factor

In summary, the following measurable indicators of each core factor are collected. Since CORE-CC is expressed into a comparison of the safe thresholds and the pressure generated by human demand, a set of methodologies for indicators estimation are described from the ends of pressure and support. For the core factors of resources and environment, the supply of resources and the capacity of environment are taken as the support, while the actual demand of resources and the discharge of pollutants in the social economic system are taken as the pressure. For the core factors of ecosystem and socio-economic, the actual ecological services provided by the ecosystem and the actual economic input provided by the socio-economic system are taken as the support, while the expected value of a specific standard or environmental quality improvement requirements is taken as the pressure.

#### Land resource

This factor is measured by indicator of suitable construction land resource. Land resources have functions of production, carrying capacity, providing raw materials, saving and value-added, etc. The CORE-CC focuses on the spatial carrying capacity function of land as the basic activity place of human beings and building base, and the analysis of its supply capacity mainly focuses on construction land. The pressure end of this indicator is the developed land use area of coral reef islands, and the support end is the area suitable for construction land. The amount of land resources suitable for construction refers to the amount of land resources whose natural conditions and socio-economic conditions are suitable for construction purposes and will not adversely affect the ecological environment after development. The calculation is mainly obtained through the ecological suitability assessment of construction land. The assessment of the ecological suitability of construction land is the process of assessing the suitability of the land for various construction uses from the perspective of ecological environmental protection. The main factors affecting the suitability of the land are selected (including land cover, fracture zone, mean gradient, and soil property). Based on the GIS tool, a multi-factors weighted scoring method is used to calculate the appropriate construction land resources. The comprehensive ecological suitability score is obtained by using the following equations:

1 $$ {C_i} = \left\{ {\begin{array}{ll}
0& \quad {{C_{ij}} = 0 }\\
{\sum\limits_{j = 1}^n {{W_j} \times {C_{ij}}} }& \quad {{C_{ij}} \ne 0}
\end{array}} \right.$$

where *C*_*i*_ is the comprehensive ecological suitability score of the *i*-th evaluation unit, *i* = 1,2,3…m; *C*_*ij*_ is the suitability grade score for the *j*-th evaluation factor of the *i*-th evaluation unit, *j* = 1,2,3…n; *W*_*j*_ is the weight of the *j*-th factor. The results of evaluation unit are classified into four levels: forbidden, limited, less suitable, or suitable. Among them, the area land of less suitable and suitable is accounting for the suitable construction land resource.

#### Water resource

This factor is measured by indicator of the available water resource that refers to the maximum amount of water resources that can be developed and used within a certain time and space without prejudice to environmental systems. The pressure end of this indicator is the annual water consumption of the total population, while the support end is the annual total available water resources, which is composed of available surface water, available ground water, trans-boundary water and wastewater reclamation. The calculation equation is as follows:

2 $$ W_{AR} = W_{G} + W_{T} + W_{RE} + W_{D} $$

where *W*_*AR*_ is the total available water resource, *W*_*G*_ is available ground water, *W*_*T*_ is water transferred in, *W*_*Re*_ is reclaimed wastewater, and W_*D*_ is desalinated sea water.

#### Water assimilation

According to the characteristics of the treatment of coral reef island pollutants, the water assimilation refers to the ability of the seawater around the coral reef island to keep the water body clean through the migration of water mass and degradation of pollutant concentration. The pressure end of this indicator is calculated according to the annual emissions from point sources (e.g., sewage treatment plants), while the support end is estimated by the water assimilative capacity proposed in the total maximum daily load (TMDL) process, to achieve compliance with the pollutant standards^73^. In this paper, inorganic nitrogen (IN) and inorganic phosphorus (IP) as the main pollutant indicators. A DHI MIKE3 software was used to establish a coupling model of hydrodynamics and water quality in the offshore waters of the coral reef islands. The movement and water quality of pollutants in the offshore waters were simulated, and the response field results reflecting the relationship between the pollutant concentration and the emission sources in the near waters were obtained. In addition, the classic linear optimization method is used to obtain the maximum total pollution load of each pollution source under the premise of meeting specific water quality standards, which is used as the water assimilation capacity^74^. It is expressed as,

3 $$ C_{i} = \sum\limits_{i = 1}^{n} {P_{ij} Q_{j} } $$

4 $$ \begin{gathered} AC = Max\sum\limits_{j = 1}^{n} {Q_{j} } \hfill \\ {\text{ Subject to }}C_{i} \le C_{i}^{s} \hfill \\ \end{gathered} $$

5 $$ Q_{j}^{l} \le Q_{j} \le Q_{j}^{u} $$

where *i* is the sea area number, *j* is the point source number, $C_{i}^{{}}$ is the pollutant concentration in the sea area *i*, $C_{i}^{s}$ is the reference standard for quality of seawater in sea area *i*, $P_{ij}^{{}}$ is pollutant response field of source j in sea area *i*, $Q_{j}$ is optimized pollutant load of source *j*, $Q_{j}^{l}$ and $Q_{j}^{u}$ are the minimum and maximum pollutant load limits of point source *j*, *AC* is the seawater assimilative capacity.

#### Terrestrial ecosystem services

This factor is measured by indicator of Sum LAI. Leaf area index (LAI) is one of the most widely used parameters in ecological science, which is used to describe the ecological health of natural ecosystems. Most of the ecological service functions of terrestrial natural ecosystems originate from metabolic processes of plants or related processes, including photosynthesis, transpiration, and material circulation, and the leaf area plays a decisive role to a large extent^75^. Therefore, the LAI can approximately represent the ecological service function. For a region, the sum of average LAI for all types of land cover (Sum LAI) is often used to reflect the level of ecosystem services^76^. The pressure end of this indicator is the expected value of the Sum LAI to meet the functional orientation and development planning of the coral reef islands, while the support end is the actual measured value. The equation is as follows:

6 $$ L_{S} = \sum\limits_{i = 1}^{n} {L_{i} } \cdot \frac{{A_{i} }}{{A_{is} }} $$

where, *L*_*S*_ is the sum LAI value, *i* is the land cover type, *L*_*i*_ is the averaged LAI value of *i*-th land cover type, *A*_*i*_ is the area of the *i*-th land cover type, *A*_*is*_ is the sum area of land covers. The types and proportions of land cover can be obtained through remote sensing image supervision and classification combined with field investigation. The averaged LAI for each type of biome/land covers employ the empirical values^77,78^ listed in Table 2.

**Table 2:** Average LAI of land cover type (m^2^/m^2^).

Land cover	Farmland	Garden	Woodland	Grassland	Wetland	Construction land	Other land
Average LAI	3.0	3.0	5.0	2.0	6.5	0.5	1.0

#### Coral reef ecosystem services

Coral reef ecosystems provide a rich variety of direct and indirect services to humans, such as food supply, coastal protection, diving tourism, etc. By preserving coral ecosystems’ capacity to cope with disturbances, we can preserve the ability of ecosystems to provide us with the goods and services that we depend on. The coral coverage is often used as an indicator of the health and resilience of coral reefs in general, and a decline in coral coverage means reduced habitat supply and reduced service capacity^12,79^. Among which, Hard corals are reef-building and when they die the skeleton endures and creates a base for other corals to grow on. They are considered as the most important component of coral reefs because they build durable structures that provide habitat for countless coral reef organisms^80^. Furthermore, if the coral reef has a high biodiversity of plants and animals that perform different functions, the buffer capacity is usually higher^81^. Thus, this factor is measured by indicators of hard corals coverage, species number hard corals, coral reef fish density, and species number of coral reef fishes for assessment. Referring to the coral reef health status standard set by Zufikar^82^ and Sun^83^, the pressure end of these indicators is the demand value that can maintain the health of coral reef ecosystem, and the supporting end is the actual value of these indicators. These indicators are available through field surveys and remote sensing. Among them, the calculation formulas of hard corals coverage and coral reef fish density are as follows:

1. Hard corals coverage (*HCC*)
  7 $$ HCC = n \times M \times 100\% $$
  where, *n* is the number of hard corals on each survey line, and *M* is the total number of points on each line.
2. Coral reef fish density (*D*_*f*_)
  8 $$ D_{f} = \frac{N}{2LW} $$
  where, *N* is the total number of reef fish on the sample line; *L* is the total length of the sample line (m); *W* is the width of one side of the sample line (m).

#### Socio-economic supporting

This dimension contains the level of socioeconomic technology development and human willingness to pay for ecological and environmental protection. The core factors are measured by the indicators of GDP per capita, environmental protection investment and R&D expenditures. The support end of these indicators is determined by actual values for the year and can be obtained from local statistics. The pressure end of GDP per capita is calculated by referring to the Environmental Kuznets curve^84^, local economic development indicators and environmental quality^85^. The pressure end of environmental protection investment and R&D expenditures is calculated according to the expected value set by the national or local government development plan^16,86^^.^.

### Indicators system of assessing CORE-CC

The essence of core-cc assessment is to judge whether the pressure brought by social development is within the support capacity provided by reef island system. Along this line of thought, based on the above-mentioned CORE-CC concept and connotation, a comprehensive assessment indexes system was established (Table 3). The characteristic of this indicator system is that there is a one-to-one correspondence between the support indicator and the pressure indicator. The support indicator value can be regarded as the threshold value of the pressure indicator, which can easily evaluate whether the regional development is overloaded and the degree.

**Table 3:** Indicators system for CORE-CC.

Objective (c)	Dimensions (c_i_)	Primary		
		Support indicator (s_ij_)	Pressure indicator (p_ij_)	Unit
CORE-CC	Resources supply	Suitable construction land resource	Construction land	km^2^
		Available water resource	Water consumption	t/a
	Environmental assimilation	Water assimilation capacity	Pollutant discharge	t/a
	Ecosystem services	Sum LAI	Target Sum LAI	m^2^/m^2^
		Hard corals coverage	Target hard corals coverage	%
		Species number of hard corals	Target species number of hard corals	-
		Coral reef fishes density	Target coral reef fishes density	Fish/m^2^
		Species number of coral reef fishes	Target species number of coral reef fishes	–
	Socio-economic supporting	GDP per capita	Target GDP per capita	Yuan
		Environmental Protection Investment	Target environmental Protection Investment	%
		R&D Expenditures	Target R&D Expenditures	%

### Assessment of CORE-CC

#### Assessment of the core indicators

Once the paired pressure/support indicators and their values are determined, they can be compared directly. By determining the ratio of the remaining quantity between the index pairs, it is determined whether the carrying state is overloaded. The equation is as follows:

9 $$ d_{ij} = \frac{{p_{ij} - s_{ij} }}{{s_{ij} }} = 1 - \frac{{p_{ij} }}{{s_{ij} }} $$

where s_*ij*_ is the support indicator, and p_*ij*_ is the pressure indicator. The principle of assessment is the following. For *d*_*ij*_ > 0, surplus occurs when support capable of meeting the pressure, which represents as the degree of surplus. For *d*_*ij*_ = 0, the pressure has reached the support capacity threshold, and no more pressure should be placed on this indicator. For *d*_*ij*_ < 0, deficit occurs when pressure exceeds support, which represents the degree of transgression.

#### Assessment of the CORE-CC and its dimension indexes

According to the theory of regional carrying capacity vector assessment proposed by Yu and Mao^49^, CORE-CC can be regarded as a vector containing four dimensions, and each dimension index can be regarded as a subvector containing several core indicators. Liu and Borthwick^56^ based on this idea, proposed a vector of carrying capacity surplus ratio model. The model first uses the vector modulus to calculate the carrying capacity surplus ratio of each dimension, then calculates the comprehensive carrying capacity surplus ratio from each dimension’s surplus ratio. Each dimension (indicator) has equal opportunities for the factors that restrict the CORE-CC, so the same weight is used for assessment. For dimension index assessment, we define the surplus ratio of the dimension index as:

10 $$ D_{i} = 1 - \sqrt {\frac{1}{{n_{i} }}\sum\limits_{j = 1}^{{n_{i} }} {(1 - d_{ij} )^{2} } } $$

where *D*_*i*_ is the surplus ratio of the *i*-th dimension, *n*_*i*_ is the number of core indicators of the *i*-th dimension index, d_*ij*_ is the same as above. For *D*_*i*_ > 0, it implies the surplus state. For *D*_*i*_ = 0, it implies the full- loaded state. Whereas for *D*_*i*_ < 0, it implies the over-loaded state.

For the CORE-CC assessment, combining vector of each dimension index to get a surplus ratio of CORE-CC index, which can be described as:

11 $$ D = 1 - \sqrt {\frac{1}{m}\sum\limits_{i = 1}^{m} {(1 - D_{i} )^{2} } } $$

where *D* is the surplus ratio of the CORE-CC, *m* is the number of dimensions (equal to 4 in the present study). For *D* > 0, the coral reef island is fully capable of carrying the pressure induced by human interference, whereas for *D* < 0, the coral reef island is over-loaded and negative to carry the pressure.

### Study area and data sources

Zhaoshu Island (ZSI) is one of the main coral reef islands of the Xisha Islands in the South China Sea. Its geographical coordinates are 16°59′N, 112°16′E, and an area of about 0.22 km^2^. It is formed by the accumulation of white coral skeletal and shell sands on a reef platform, and is about 4.4 m above sea level. There are lots of hard disk phosphorous lime soil on the island, and the vegetation is mostly Scaevola sericea. ZSI community belongs to Sang City, Hair Province, China. The residents are generally composed of fishermen, government workers and civil servants who have settled down for a long time or visited for a short time. With the gradual opening of the island's tourism policy, its tourism industry is booming and the number of tourists is increasing. Since the official establishment of Sansha municipal government in 2012, the living conditions and environment of the local residents in ZSI have been significantly improved, and the infrastructure construction of road facilities, power grid, sewer and so on is complete. With the development and utilization of coral reef islands strengthened, the pressure on the ecological environment of the ZSI is also increasing. Through the comprehensive carrying capacity assessment to provide a reference for the sustainable development of ZSI.

According to the assessment method described above, an assessment index system for the carrying capacity of the coral reefs of ZSI was established, and the carrying stage of ZSI in 2017 was evaluated and analyzed. The data of each indicator are obtained through field surveys, local government statistical yearbooks, interviews with local government staff, historical research literature, and remote sensing monitoring.

## Results

### Assessment results of core indicators

The surplus ratio of each core indicator is calculated by Eq. (9), and the carrying state and degree of each core element of the coral reef island system are determined (Table 4).

**Table 4:** Assessment of core indicator of CORE-CC in ZSI in 2017.

CORE-CC dimensions	Core indicator	Surplus ratio	Carrying state	Degree (%)
Resources supply	Land resources	0.38	Surplus	38
	Water resources	0.44	Surplus	44
Environmental assimilation	Water assimilation capacity	0.87	Surplus	87
Ecosystem services	Sum LAI	− 0.04	Over-load	4
	Hard corals coverage	− 1.45	Over-load	145
	Species number of hard corals	0.14	Surplus	14
	Coral reef fishes density	− 0.67	over-load	67
	Species number of coral reef fishes	0.14	Surplus	14
Socio-economic supporting	GDP per capita	0.04	Surplus	4
	Environmental protection investment	0.08	Surplus	5
	R&D expenditures	− 0.60	Over-load	60

On the whole, 4 of the 11 core indicators of ZSI’s CORE-CC in 2017 were overloaded. The Sum LAI, Hard corals coverage, and coral reef fish density in the ecosystem service capacity dimension are overloaded by 4%, 145%, and 67%, respectively, indicating that both land and marine ecosystems cannot provide stable and adequate ecosystem services. Among them, the marine ecosystem is particularly overloaded, with two indicators of the highest degree of overload. This indicates that ZSI's coral reef ecosystem service capacity has been severely degraded by climate change and human activities. The R&D expenditure indicator in the social support capacity dimension is 60% overloaded, indicating that under the situation of economic development, the investment in research on ZSI is relatively lagging. As a practice of the national ecological civilization reform, the ‘Ecological Island’ project implemented by the State Oceanic Administration has a positive impact on the ecological environment of ZSI, making the environmental protection investment indicator appear surplus stage. The core indicators of the resource supply dimension are all surplus. Thanks to the establishment and operation of desalination plant, the available water resource of ZSI is 105 m^3^/a and tends to have a net surplus of 44%. Although the construction intensity of civil buildings continues to increase, the suitable construction land resource remains 0.13 km^2^ with a 38% surplus. The seawater assimilation capacity indicator has the highest surplus degree, with a surplus ratio of 87%. This shows that ZSI's offshore water body (1 km buffer zone) has sufficient assimilation capacity to carry the discharge of IN and IP pollutants, and can ensure that the water quality meets the national optimal water quality standard.

### Comprehensive assessment results of CORE-CC

**Figure 2:**
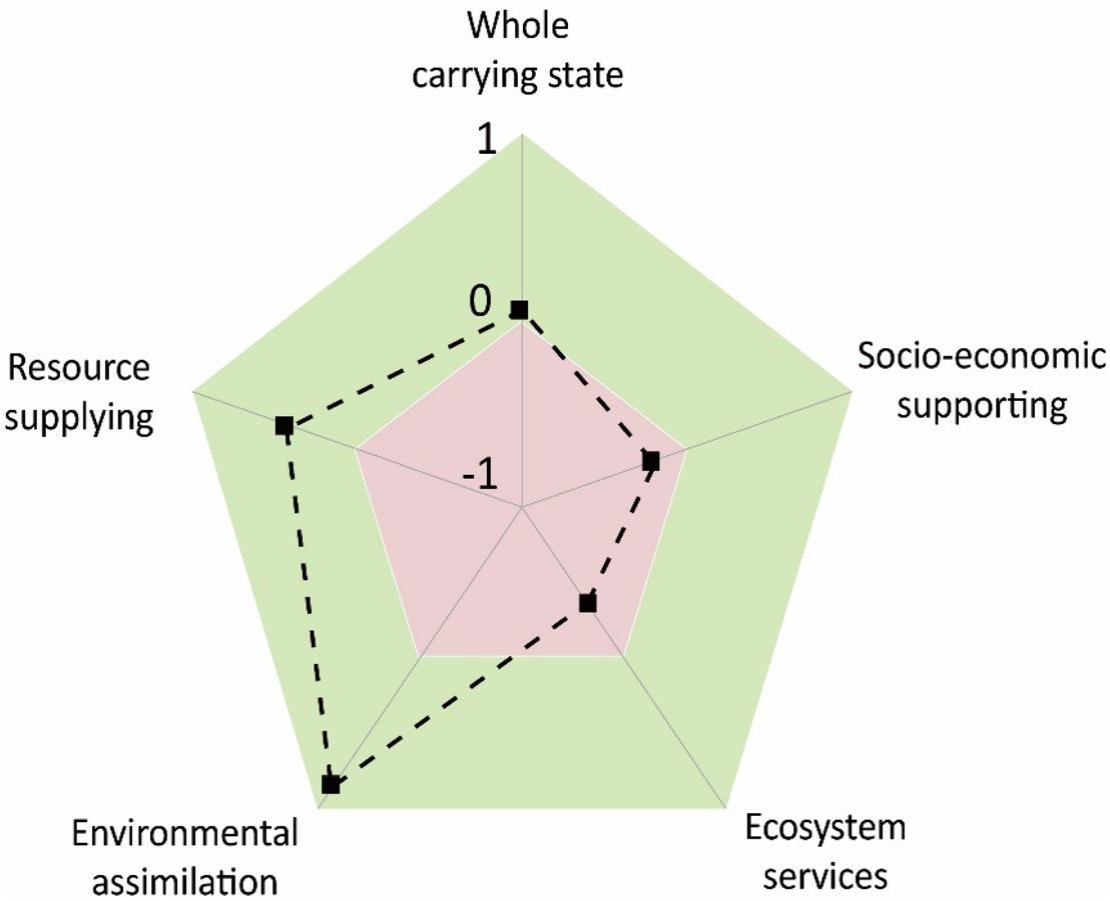
Assessment of CORE-CC (whole carrying state) and its each dimension's carrying state for ZSI in 2017.

Based on the analysis of the core indicators, a vector of carrying capacity surplus ratio model is used for the comprehensive assessment of CORE-CC to determine the comprehensive carrying stage and degree of each dimension and the entire coral reef islands system (Fig. 2). In general, ZSI’s whole carrying state in 2017 is in a surplus state, with a degree of 5%, which is close to the limit of CORE-CC. The inherent capacity has been almost exhausted by human needs, which indicates that the operation of the ZSI system is not optimistic. Although the dimensions of resource supply and environmental assimilation are in a large surplus state, the overload of other dimensions has a serious negative impact on the overall capacity. This is reflected in the lack of ecosystem service capacity caused by the unhealthy ecosystem and the lack of social support capacity caused by the lagging behind of protective scientific research investment in economic development. These findings indicate that the state of marine and terrestrial ecosystems and the scarcity of relating fundamental research play a key role in ZSI's CORE-CC, which should have drawn major concerns.

## Discussion

### Discussion on the case demonstration results

The CORE-CC model proposed in this paper indicates the new connotation of coral reef islands comprehensive carrying capacity from the four dimensions of resources, environment, ecosystem and socio-economic. Moreover, the core indicators are selected according to the characteristics of each dimension, which is conducive to quickly determining the carrying status. The results of the comprehensive assessment highlight the overall pressures on the coral reef island, which can help local decision-making and provide information for the coral reef island’s sustainable development. The case study of ZSI showed that the model can be applied to local contexts and that is potentially useful in practice as a guide for planning. There is still a large surplus in the dimension of resource supply and environmental assimilation of ZSI's carrying capacity, but this is only because ZSI is in the primary stage of construction and the optimistic situation brought by the small population and industrial scale. At the same time, if the assessment standard of environmental assimilation capacity is changed to coral healthy water quality limit, its carrying capacity will be greatly reduced. Therefore, although the carrying capacity of resources and environmental is in surplus state, it does not mean that resources can be squandered and pollution can be discharged at will. Due to the common interference of human activities and natural changes, factors that threaten ecological resilience and health set the tone for the regional decline in coral reef ecosystem service^87^, so we are not surprised that ZSI’s ecosystem service carrying state are overloaded. As an administrative division newly established in 2012, ZSI experienced a lot of reclamation activities and excessive pollution caused by intensive shipping in the initial stage of construction, which may lead to the degradation of coral reef ecosystem services. Although the ecological restoration of ZSI has been ongoing, at least it has not yet returned to the ideal state.

In terms of socio-economic supporting, due to the lack of significant economic benefits and good investment allocation of ZSI, the positive feedback of ecological environment is hindered. At present, ZSI focuses on the physical carrier of human survival, and gradually implements basic functions in a human-controlled manner. In the realization process of functional zones under the guidance of policies, due to the open source of production materials and construction capital, the CORE-CC of ZSI has a relatively large elastic range, and the autonomous carrying capacity is relatively weak. Maintaining a good foundation of care conditions and establishing appropriate regulations and policies for risk control are important prerequisites for enhancing the self-sufficiency of coral reef island resources and ensuring their sustainability. In terms of marine eco-civilization practice proposed by 18th CPC National Congress, timely release of priority measures to ease supply pressure, strengthen ecological restoration, and promote the optimal allocation of resources and services to ensure the sustainable development of ZSI.

### Discussion on the uncertainties and future work

The comparative analysis of pressure-support indicators may help to understand the concept and mechanism of carrying capacity, but it also introduces the uncertainty of the quantification of carrying capacity. The CORE-CC model essentially takes the pressure-support index comparison result d (see Eq. 9) equal to zero as the critical point to judge the carrying status of each element. Since most of the pressure or support group parameters are estimated through functions and data sets, the data will inevitably be ambiguous, which leads to uncertainty in the comparison between the results and the critical point. In this case study, except for a few indicators data (e.g., GDP per capita) released by government agencies that are definite values, the estimated data of most other indicators may be uncertainty range or confidence interval (e.g., Water Resources, Sum LAI, etc.). Our treatment is to choose the average value. This is a conventional method, but it also brings uncertainty to the comparison results^88^, especially when the value range of the pressure end and the support end are close. Therefore, we recommend setting a certain amount of uncertainty buffer (such as 10%) as warning zone near the critical point, which means that when the result is greater than 0 and less than 10%, the carrying capacity is considered to be in a warning state. This allows society time to react to early warning signs approaching the critical point and the subsequent possible overload risk^89^, instead of entangled in the uncertainty of the carrying capacity state. Another type of uncertainty relates to indicator weights. Weights can indirectly integrate the views of stakeholders^90^ or experts' opinions on local policy priorities^91^, and are often used as measures of perceived importance of the subgroup to the system^92^. We used equal weights due to a lack of knowledge of the study area. However, in practice, the importance of various carrying capacity factors to coral reef islands with different economic structures, stakeholders and development policies may not be the same^93,94^. In this regard, we encourage development and use of weights for localised assessments to maintain the usefulness of the model in different types of coral reef islands.

We strive to have a comprehensive and versatile model for coral reef islands, but the core factors and indicators selected in this article may not cover all of them. The indicators of each dimension may be more abundant in future research, such as increasing energy supply and fishery supply. Considering the difficulties encountered in assessing the correct match between pressure and support and establishing standard thresholds, the representation of ecosystem service capacity needs to be further improved. In view of these problems, it is necessary to further explore ways to enrich the CORE-CC framework, propose an acceptable indicators and standard thresholds list, and dynamically monitor the resource consumption and ecological environment status related to social development.

## Conclusions

According to the sustainable development needs of coral reef islands and the essence of the concept of carrying capacity, this paper proposes a conceptual model of coral reef island carrying capacity (CORE-CC) from the perspective of pressure-support. The model includes four dimensions: resource supply, environmental assimilation, ecosystem services and socioeconomic supporting. According to the characteristics of the coral reef islands, the core factors and indicators of each dimension are selected and the corresponding assessment index system of "pressure-support" is constructed. Using the pressure/support comparative analysis method, the comprehensive assessment of carrying capacity was conducted with ZSI as the case study. The results show that the ZSI’s CORE-CC in 2017 still maintained a carrying capacity surplus ratio of 5%. Among them, the resource supply capacity and environmental assimilative capacity are in a good carrying state, and the surplus ratios were 41% and 87%, respectively. The ecosystem service capacity and social support capacity are both overloaded, with overload degrees of 36% and 20%, respectively. This is also the key factors that lead to ZSI’s CORE-CC nearing full load, and it must be targeted for improvement.

Case studies show that the CORE-CC model is suitable for measuring the carrying capacity of coral reef islands. The measurement results not only show the carrying states of each dimension, but also provide a data basis for resource balance, total amount control and ecological construction. This will help provide a support for decision-making on the scale, structure, and layout of coral reef islands development.
