# Author Correction: Development of a comprehensive assessment model for coral reef island carrying capacity(CORE-CC)

**DOI:** 10.1038/s41598-021-95871-1

**Published:** 2021-08-10

**Authors:** Ming Chen, Fenzhen Su, Fei Cheng, Yu Zhang, Xuege Wang

**Affiliations:** 1 State Key Laboratory of Resources and Environmental Information System, Institute of Geographic Sciences and Natural Resources Research, Chinese Academy of Sciences, Beijing, 100101 China; 2 College of Resources and Environment, University of Chinese Academy of Sciences, Beijing, 100049 China; 3 School of Geography and Ocean Sciences, Nanjing University, Nanjing, 210023 China

Correction to: *Scientific Reports* 10.1038/s41598-021-83481-w, published online 16 February 2021

The original version of this Article contained an error in Affiliation 2, which was incorrectly given as ‘Collaborative Innovation Center for the South China Sea Studies, Nanjing University, Nanjing, 210023, China’. The correct affiliation is listed below:

College of Resources and Environment, University of Chinese Academy of Sciences, Beijing 100049, China.

The original Article has been corrected.

